# HNN-core: A Python software for cellular and circuit-level interpretation of human MEG/EEG

**DOI:** 10.21105/joss.05848

**Published:** 2023-12-15

**Authors:** Mainak Jas, Ryan Thorpe, Nicholas Tolley, Christopher Bailey, Steven Brandt, Blake Caldwell, Huzi Cheng, Dylan Daniels, Carolina Fernandez Pujol, Mostafa Khalil, Samika Kanekar, Carmen Kohl, Orsolya Kolozsvári, Kaisu Lankinen, Kenneth Loi, Sam Neymotin, Rajat Partani, Mattan Pelah, Alex Rockhill, Mohamed Sherif, Matti Hamalainen, Stephanie Jones

**Affiliations:** 1 Athinoula A. Martinos Center for Biomedical Imaging, Massachusetts General Hospital, Boston, MA, USA; 2 Department of Neuroscience, Brown University, Providence, RI, USA; 3 Robert J. and Nancy D. Carney Institute for Brain Science, Brown University, Providence, RI, USA; 4 Department of Clinical Medicine, Aarhus University, Aarhus, Denmark; 5 Bassett Medical Center, Cooperstown, NY, USA; 6 Department of Psychological and Brain Sciences, Indiana University Bloomington, Bloomington, IN, USA; 7 Department of Biomedical Engineering, University of Miami, Coral Gables, FL, USA; 8 Department of Psychiatry and Behavioral Health, Penn State Milton S. Hershey Medical Center, Penn State College of Medicine, Hershey, PA, USA; 9 Warren Alpert Medical, Brown University, Providence, RI; 10 Department of Psychology, University of Jyväskylä, Jyväskylä, Finland; 11 Jyväskylä Centre for Interdisciplinary Brain Research, University of Jyväskylä, Jyväskylä, Finland; 12 Department of Radiology, Harvard Medical School, Boston, MA, USA; 13 Department of Molecular and Cell Biology; Innovative Genomics Institute, University of California, Berkeley, Berkeley, CA, USA; 14 Center for Biomedical Imaging and Neuromodulation, Nathan S. Kline Institute for Psychiatric Research, Orangeburg, NY, USA; 15 Department of Psychiatry, New York University Grossman School of Medicine, New York, NY, USA; 16 Department of Computer Science and Engineering, National Institute of Technology Karnataka, Karnataka, India; 17 Florida State University, Tallahassee, FL, USA; 18 Department of Human Physiology, University of Oregon, Eugene, OR, USA; 19 Department of Psychiatry and Human Behavior, Brown University, Providence, RI, USA; 20 Rhode Island Hospital, Providence, RI, USA; 21 Department of Neuroscience and Biomedical Engineering, Aalto University, Espoo, Finland

## Abstract

HNN-core is a library for circuit and cellular level interpretation of non-invasive human magneto-/electro-encephalography (MEG/EEG) data. It is based on the Human Neocortical Neurosolver (HNN) software (Neymotin et al., 2020), a modeling tool designed to simulate multiscale neural mechanisms generating current dipoles in a localized patch of neocortex. HNN’s foundation is a biophysically detailed neural network representing a canonical neocortical column containing populations of pyramidal and inhibitory neurons together with layer-specific exogenous synaptic drive (Figure 1 left). In addition to simulating network-level interactions, HNN produces the intracellular currents in the long apical dendrites of pyramidal cells across the cortical layers known to be responsible for macroscopic current dipole generation.

HNN-core reproduces the workflows and tutorials provided in the original HNN software to generate commonly observed MEG/EEG signals including evoked response potentials (ERPs) and alpha (8-10 Hz), beta (15-30 Hz), and gamma (30-80 Hz) rhythms. HNN-core enables simultaneous calculation and visualization of macro- to micro-scale dynamics including MEG/EEG current dipoles, local field potential, laminar current-source density, and cell spiking and intrinsic dynamics. Importantly, HNN-core adopts modern open source development standards including a simplified installation procedure, unit tests, automatic documentation builds, code coverage, continuous integration, and contributing guidelines, supporting community development and long-term sustainability.

## Statement of need

HNN-core addresses a key need in the fields of computational and experimental neuroscience by providing an extensively documented application programming interface (API) that allows both novel and advanced users to run biophysically-principled neural network simulations out-of-the-box with a few lines of code. HNN-core modularizes the model components originally introduced by HNN and its associated graphical user interface (GUI) and provides an interface to modify it directly from Python. This has allowed for significant expansion of the HNN functionality through scripting, including the ability to modify additional features of local network connectivity and cell properties, record voltages in extracellular arrays, and more advanced parameter optimization and batch processing. A new web-based GUI has been developed as a thin layer over the Python interface making the overall software more maintainable.

## HNN-core implements a biophysically detailed model to interpret MEG/EEG primary current sources

MEG/EEG are the two electrophysiological methods to non-invasively study the human brain. They have been used in developing biomarkers for healthy and pathological brain processes. Yet, the underlying cellular and circuit level generators of MEG/EEG signals have been difficult to infer. This detailed understanding is critical to develop theories of information processing based on these signals, or to use these techniques to develop new therapeutics. Computational neural modeling is a powerful technique to hypothesize the neural origin of these signals and several modeling frameworks have been developed. Since MEG/EEG recordings are dominated by neocortical sources, all models developed so far simulate neocortical activity, but they are developed with different levels of biophysical detail and correspondingly different use cases. The level of detail in HNN’s pre-tuned network models and the workflows developed to study ERPs and low frequency oscillations are unique within the field of MEG/EEG modeling. One class of models known as neural mass models (NMMs) uses simplified representations to simulate net population dynamics, where hypothesized connectivity among neural “nodes” can be inferred from recordings. The Virtual Brain Project (Sanz Leon et al., 2013) and Dynamic Causal Modeling from the SPM software (Friston et al., 2003; Litvak et al., 2011) are prominent examples of software that implement NMMs. While NMMs are computationally tractable and advantageous for studying brain-wide interactions, they do not provide detailed interpretation of cell and circuit level phenomena underlying MEG/EEG. The primary electrical currents that create MEG/EEG sensor signals are known to be oriented along the long and spatially aligned cortical pyramidal neuron dendrites, and their direction corresponds to that of the intracellular current flow (Hämäläinen et al., 1993). For a detailed discussion see Neymotin et al. (2020). Further, source localization methods such as minimum-norm estimate (MNE) calculate the primary currents (assuming constraints defined by the technique (Gramfort et al., 2013)). As such, models created to study the cell and circuit origin of these signals are designed with detailed pyramidal neuron morphology and physiology, and are often embedded in a full neocortical column model. HNN is one such detailed neocortical column model (Neymotin et al., 2020), and other examples have been employed using the software LFPy (Lindén et al., 2014). LFPy simulates fields and currents from detailed models of pyramidal neurons and networks (Lindén et al., 2014). LFPy does not support a specific neocortical model or workflows/parameter inference to study particular signals of interest. Rather, it provides multi-use Python scripts that can be integrated into any NEURON based neural model containing multi-compartment pyramidal neurons. A unique feature of HNN is its workflows for interacting with the template neocortical model through layer-specific activations to study ERPs and low frequency brain rhythms. HNN also enables direct comparison between simulation output and the waveforms of estimated sources in the same units of measure and supports parameter inference. HNN-core was created to maintain all of the functionality of the original HNN software with additional utility (described below) and a well-defined, well-tested and documented API. Its adoption of open source development standards, including a simplified installation procedure, unit tests, automatic documentation builds, code coverage, and continuous integration, enables community development and long-term sustainability.

## HNN-core facilitates reproducibility and computationally expensive workflows

Scripting in HNN-core greatly expands the software utility particularly for large research projects where reproducibility and batch processing is of key importance. The scripted interface allows multi-trial simulations enabling the use of computationally expensive parameter optimization algorithms and parameter sweeps using parallel processing on computer clusters. HNN scripting also facilitates the creation of publication-quality figures and advanced statistical analysis. Further, the software can be integrated with existing scripted workflows, such as those developed in MNE-Python (Gramfort et al., 2013), a well-established MEG/EEG signal processing and source localization software, enabling source localization and circuit interpretation in just a few lines of code (see a tutorial of this in the HNN-core documentation).

## Notable features of HNN-core

HNN-core functionality supports advanced simulations through scripting that are not currently possible in the GUI including:

- the ability to record extracellular local field potentials from user defined positions, as well as voltages and synaptic currents from any compartment in the model;
- the ability to modify all features of the morphology and biophysical properties of any cell in the network;
- an API that enables complete control of cell-cell and drive-cell connectivity in the network;
- an API that allows for flexibility in defining the exogenous layer-specific drive to the neocortical network;
- the ability to choose from multiple template models based on previous publications (e.g., jones_2009_model( ) (Jones et al., 2009), law_2021_model( ) (Law et al., 2022), and calcium_model( ) adapted from (Kohl et al., 2022));
- built-in ERP optimization functionality designed for faster convergence;
- the choice of two parallel backends for either parallelizing across cells to speed up individual simulations (MPI), or across trials to speed up batches of simulations (Joblib).

HNN-core code has also enabled the creation of a new and improved web-based GUI based on ipywidgets (Jupyter widgets community, 2015) and voila (Voilá community, 2019) that can be run remotely with port forwarding.

All of the code associated with HNN-core has been extensively documented at multiple levels, including an API describing basic functions/parameters and examples of use for hypothesis generation and/or testing. Specifically, we distribute tutorials that mimic the original GUI tutorial workflows using HNN-core functions, with commentary on the known biophysical mechanisms of these signals.

## Use cases and quick example code of running a simulation

As summarized above, HNN-core reproduces the workflows and tutorials provided in the original GUI driven HNN software designed to investigate the origin of commonly observed MEG/EEG signals. The HNN-core tutorials include examples of how to simulate ERPs, as well as low frequency rhythms such as alpha (8-10 Hz), beta (15-30 Hz), and gamma (30-80 Hz). The tutorials also include an example of directly comparing simulations to real data (i.e., the median nerve evoked response). We also provide short and targeted “How to” examples that describe how to use specific functionality, such as plotting firing patterns, or recording extracellular LFPs.

In practice, users learn how to study the multi-scale origin of ERPs and low frequency oscillations by first following the tutorials in the HNN-GUI, and then recapitulating these tutorials in HNN-core. The tutorials provide an interactive investigation that gives intuition on how exogenous drives and other parameters in the model impact the outputs of the simulations. From there, users can test hypotheses about what parameters or sets of parameters need to be adjusted to account for their recorded data by directly comparing simulation output to data. Automated parameter inference can be performed to optimize parameters to produce a close fit (i.e., small root mean squared error) to current source ERPs, and more advanced parameter inference methods are in development.

**Figure 1:**
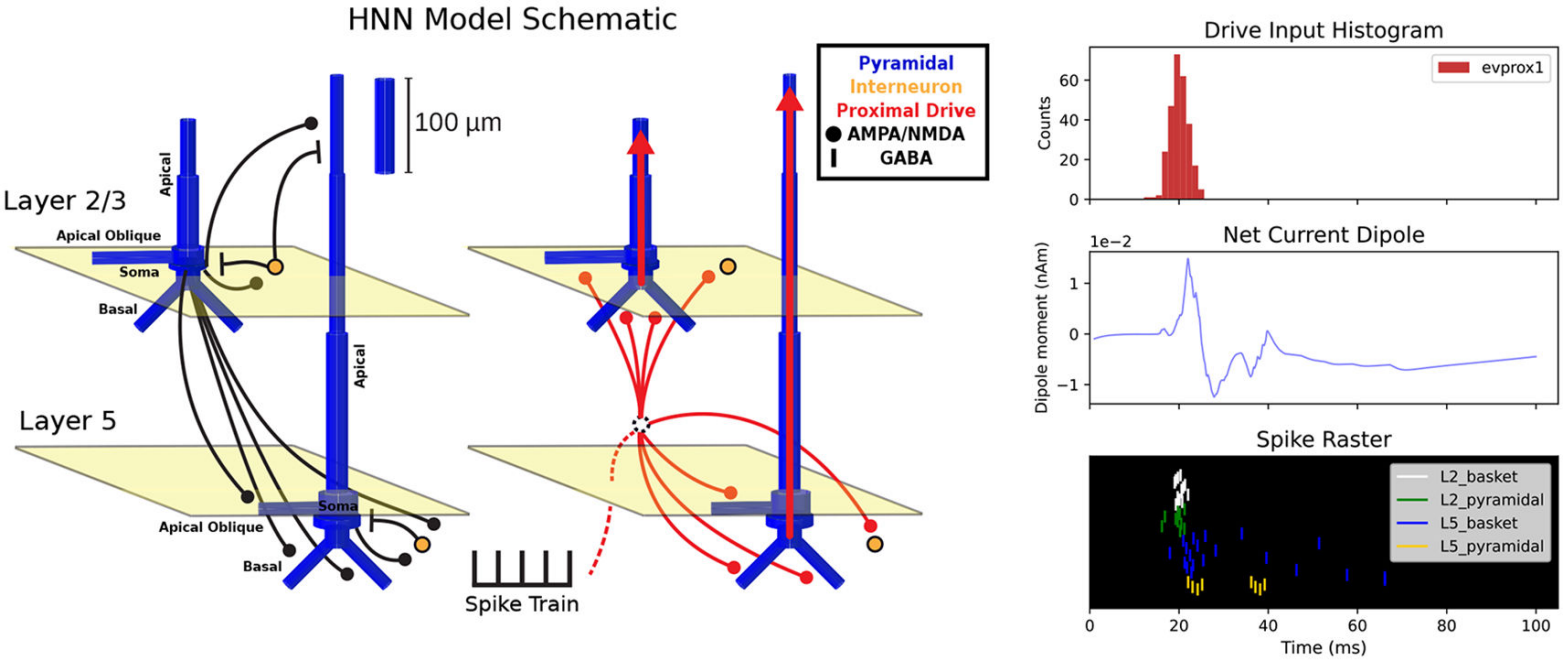
**Left:** Reduced schematic of HNN model detailing the cell types, layer-specific synaptic connectivity structure, and locations of proximal drive synapses. The default size of the full network is a grid of 100 pyramidal neurons, and 35 inhibitory neurons, synaptically connected in each layer. Figure adapted from Neymotin et al. (2020). **Right:** Plots of the network and simulated results can be generated using the HNN-core visualization API. The drive input histogram with net.cell_response.plot_spikes_hist(), the net current dipole with plot_dipole(dpl), and the spike raster with net.cell_response.plot_spikes_raster().

HNN-core has minimal dependencies which allows for effortless installation using the pip Python installer. In addition to NumPy (Harris et al., 2020), SciPy (Virtanen et al., 2020), and Matplotlib (Hunter, 2007) common in most libraries in the scientific Python stack, HNN-core uses NEURON (Hines & Carnevale, 1997) for the cell and circuit modeling. Here, we demonstrate how the HNN-core interface can be used to quickly simulate and plot the net cortical dipole response to a brief exogenously evoked drive representing “feedforward” thalamocortical input (Figure 1 right). This input (referred to as ‘evprox1’) effectively targets the proximal dendrites of the pyramidal neurons in L2/3 and L5, using the template neocortical model as in Jones et al. (2009) (Figure 1 left). Note that this simulation is not addressing a specific scientific question, and is simply an educational example.


**from** hnn_core **import** jones_2009_model, simulate_dipole
*# 1) Create the network model*
net = jones_2009_model()
*# 2) Define weights and delay times of inputs to network*
weights_ampa = {'L2_basket': 0.09, 'L2_pyramidal': 0.02,
                'L5_basket': 0.2, 'L5_pyramidal': 8e-3}
synaptic_delays = {'L2_basket': 0.1, 'L2_pyramidal': 0.1,
                   'L5_basket': 1.0, 'L5_pyramidal': 1.0}
*# 3) Attach inputs to to network*
net.add_evoked_drive(name='evprox1', mu=20.0, sigma=2.0, numspikes=1,
                     weights_ampa=weights_ampa, location='proximal',
                     synaptic_delays=synaptic_delays)
*# 4) Run simulation and plot results*
dpl = simulate_dipole(net, tstop=100.0)


## Ongoing research using HNN-core

The scripted interface of HNN-core has enabled the development of advanced parameter inference techniques (Tolley et al., 2023) using Simulation-Based Inference (Tejero-Cantero et al., 2020). It has been used in Thorpe et al. (2021) to propose new mechanisms of innocuous versus noxious sensory processing in the primary somatosensory neocortex. Lankinen et al. (2023) have used HNN-core to study crossmodal interactions between auditory and visual cortices. They performed group analysis on multiple subjects along with optimization and nonparametric statistical testing. Additionally, Szul et al. (2023) used it for understanding features of beta bursts in motor cortex and Fernandez Pujol et al. (2023) to study auditory perception.

Overall, HNN-core provides an expandable and sustainable Python-based software package that can help advance understanding of the cellular and circuit mechanisms of MEG/EEG signal generation and ultimately lead to new neuroscience discoveries.
